# Modeling Chronic Dacryocystitis in Rabbits by Nasolacrimal Duct Obstruction with Self-Curing Resin

**DOI:** 10.1155/2017/3438041

**Published:** 2017-06-22

**Authors:** Kai Hou, Tao Ai, Rong Liu, Nan Xiang, Jing Jin, Weikun Hu, Ban Luo

**Affiliations:** ^1^Department of Plastic Surgery, Tongji Hospital, Tongji Medical College, Huazhong University of Science and Technology, Wuhan 430030, China; ^2^Department of Radiology, Tongji Hospital, Tongji Medical College, Huazhong University of Science and Technology, Wuhan 430030, China; ^3^Department of Ophthalmology, Tongji Hospital, Tongji Medical College, Huazhong University of Science and Technology, Wuhan 430030, China; ^4^Outpatient Office, Yichang Central People's Hospital, The First Clinic College of Three Gorges University, Yichang 443000, China

## Abstract

We established a chronic dacryocystitis model by injecting of 0.05, 0.1, and 0.15 ml self-curing resin via the lacrimal punctum in rabbits. Animals were randomized into four groups (*n* = 11 animals/group). The control group received 0.15 ml normal saline. Within three months postinjection, epiphora and eye discharge were observed. At the 90th day postlacrimal passage irrigation, CT dacryocystography was performed to find changes in the lacrimal image, and hematoxylin and eosin staining was made to identify pathological changes of the lacrimal sac. Three months postinjection, the rabbits in control group and those who received 0.05 and 0.1 ml self-curing resin failed to develop chronic dacryocystitis. However, 8/11 (72.7%) rabbits those received 0.15 ml self-curing resin were symptomatic and showed complete reflux in lacrimal passage irrigation, indicating the obstruction of the nasolacrimal duct. CT dacryocystography showed that the obstruction was present only in the animals with chronic dacryocystitis. Pathological examinations of chronic dacryocystitis also revealed significantly inflammatory changes, such as mucus epithelium thickening, irregular papillary proliferation, and submucosal fibrous deposition. Local injection of 0.15 ml self-curing resin can induce permanent obstruction of the nasolacrimal duct in rabbits and establish a model of chronic dacryocystitis.

## 1. Introduction

**Figure 1:**
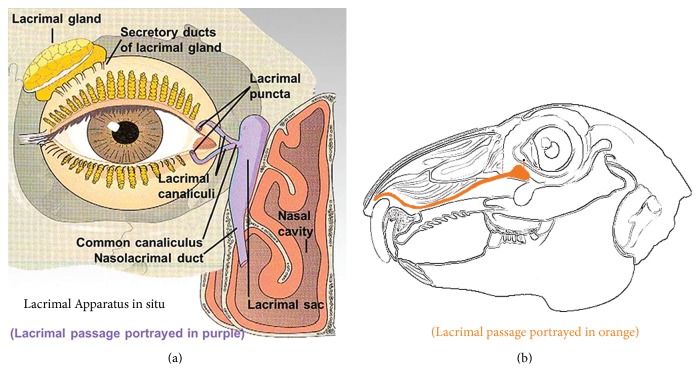
Schematic illustration of lacrimal passage in human (a) and rabbits (b).

Lacrimal passage obstruction is a common and frequently occurring eye disorder in which there appears to be an obstruction in specific parts of the lacrimal passage (Figure 1(a)). It can occur in the lacrimal canaliculi, common canaliculus and/or nasolacrimal duct, which are usually referred to an eye specialist as epiphora symptoms. In addition, nasolacrimal duct obstruction (NLDO) may also induce chronic dacryocystitis, resulting in epiphora accompanied with mucous or purulent secretion [1, 2].

Lacrimal passage obstruction seriously affects the ocular comfort and quality of life of patients. Meanwhile, chronic dacryocystitis may also induce acute dacryocystitis, keratitis, orbital cellulitis, and other severe ocular complications [1, 3, 4]. In addition, chronic dacryocystitis is a contraindication for surgical intervention in the settings of cataract, glaucoma, and other intraocular surgeries [4, 5]. With improved quality of life, prevention and treatment of lacrimal passage obstruction has emerged as a key focus of current research efforts in the field of basic and clinical ophthalmology, and this is particularly evident for in the context of NLDO.

Currently, surgery is the major mode of therapy for treating lacrimal passage obstruction; however, the success rate of this intervention remains unsatisfactory [6]. It has been previously reported that for complex congenital NLDO in infants older than 1 year of age, the success rate of probing lacrimal passage within 6 months is only 47.4% [7]. Dacryocystorhinostomy (DCR) was reported to offer good success rates around 90% with a safety profile in treating NLDO [8]. Yet, it was a concern that 5–10 percent of adult NLDO patients still retain different degrees of epiphora symptoms following successful DCR treatment [9].

Therefore, it is particularly important to systematically investigate pathophysiological mechanisms that are involved in lacrimal passage obstruction, especially in the setting of NLDO, and to seek out novel improved treatment protocols for this condition. Unfortunately, modeling of lacrimal passage obstruction has been less frequently reported, and there still lacks a reliable and well-established animal model, which greatly limits potential studies on determining the pathophysiological mechanisms responsible for lacrimal passage obstruction and subsequent chronic dacryocystitis [10–12].

In this current study, room temperature-curing denture base resin (self-curing resin) was injected into rabbits through the lacrimal punctum, with the intention of preparing a maneuverable and reproducible model of NLDO. This approach would subsequently permit pathologic analysis and therapeutic exploration of chronic dacryocystitis as well as other lacrimal passage diseases.

## 2. Materials and Methods

### 2.1. Animal Modeling

Experimental animals were provided by the Animal Center of Huazhong University of Science and Technology. Forty-four healthy male or female New Zealand rabbits were selected with a weight of 2.0–2.5 kg, age of 3-4 months. They were bred with 100 g/d special rabbit chow and 500 ml/d water intake ad libitum. All animal experiments in the study were approved by the animal care and use committee in the Huazhong University of Science and Technology.

Rabbits were subjected to flashlight observation and the lacrimal sac squeeze test to exclude ocular diseases as well as head and facial diseases that included dacryocystitis, eyelid anomaly, conjunctivitis, eyeball anomaly, and ocular neoplastic disease. Then, a 5 ml syringe was connected to the lacrimal cannula needle, which was inserted into the lacrimal canaliculus to a depth of 3 mm via the lacrimal punctum for lacrimal passage irrigation. During this procedure, an absence of liquid reflux and outflow from the nasal cavity indicated a lack of lacrimal passage obstruction.

The 44 rabbits were divided into 4 groups according to a random number table, each consisting of 11 animals. The rabbits were anesthetized by injecting a mixture of ketamine hydrochloride (China National Pharmaceutical Group) at 2 ml/0.1 g and chlorpromazine hydrochloride (China National Pharmaceutical Group) at 2 ml/50 mg and at a ratio of 1 : 1 into the hind leg muscles at a combined dose of 1-2 ml/kg body weight. Then, an appropriate amount of mixed resin was drawn using a 1.0 ml disposable syringe, which was connected to a number 9 lacrimal cannula needle and inserted into the lacrimal canaliculus to a depth of 3-4 mm through the right lacrimal punctum (Figure 1(b)). This procedure permitted controlled slow injection of the mixed resin, with doses of 0.05, 0.1, and 0.15 ml in three groups of animals, respectively. Another group was considered as the control group and received an injection of 0.15 ml normal saline only.

After receiving the injection, periocular phenotype and the presence of epiphora were observed every day. The rabbits were subjected to lacrimal passage irrigation at the 7th, 30th, 60th, and 90th day postinjection. At the 90th day postinjection, all experimental rabbits then received CT dacryocystography to clarify the location of the lacrimal passage obstruction site and the extent of the obstruction. After that, animals were sacrificed by air embolism to the auricular vein, which was then followed by pathological examination of the lacrimal sac.

### 2.2. Preparation of Self-Curing Resin

Self-curing resin was the mixture of dental base acrylic resin liquid (mainly methyl methacrylate, also contains methacrylic acid and methyl ester) and dental base acrylic resin powder (mainly poly methyl methacrylate). At room temperature of 25°C, dental base acrylic resin liquid and dental base acrylic resin powder (Wuhan Dingguo Biological Technology Co. Ltd., China) were mixed (at a mass ratio of 2 : 1) for about 30 s to produce a pasty resinous polymer. At this time, the polymer particles were tightly squeezed and presented a good flowability, such that the test article did not easily adhere to the instrument and was relatively easy to handle for drawing up into the instrument and subsequent intralacrimal injection.

### 2.3. CT Dacryocystography

The rabbits were anesthetized exactly as described above. Then, 3 ml of a mixture of meglumine diatrizoate (Bayer HealthCare, Germany) and medical sodium hyaluronate gel (Hainault) (at a volumetric ratio 1 : 1) was quickly injected into the lacrimal passage through the lacrimal punctum using a 5 ml disposable syringe and a number 9 lacrimal cannula. Subsequently, all rabbits underwent orbital CT scans using a Light Speed 16-sliced spiral CT scanner (GE Healthcare, USA), with a slice thickness of 0.625 mm. Then, all scanned images were reconstructed using maximal intensity projection (MIP) before measurements.

### 2.4. Pathological Examination of Lacrimal Sac

A probe was inserted into the lacrimal sac from the lacrimal punctum to determine the precise location of the lacrimal sac. Then, under the surgical microscope (Suzhou 66 Equipment Ltd., China), successive incisions were made to the skin, subcutaneous tissues were isolated, and the lacrimal sac was exposed, which was the visible “teardrop” dilated cavity. Subsequently, the harvested lacrimal sac tissues were placed into a specimen bottle and fixed in 4% formalin solution for 24 hr. Then, the tissues were paraffin-embedded by routine procedures and sliced using a 2235 rotatory microtome (Leica RM, Germany), followed by hematoxylin and eosin (H&E) staining procedures and observation under an optical microscope (Olympus, Japan), with a final magnification of 100x and 1000x, respectively.

### 2.5. Statistical Analysis

Statistical analyses were performed with SPSS statistical software version 18.0 (SPSS Inc., Chicago, IL, USA). Normally distributed data are presented as mean ± standard deviation (SD) of at least three independent experiments. Statistical significance was evaluated by one-way analysis of variance (ANOVA) with the Student-Newman-Keuls (SNK) test for post hoc analysis. A two-tailed alpha value of *P* < 0.05 was considered statistically significant.

## 3. Results

### 3.1. Modeling Results

Criteria for successful chronic dacryocystitis modeling were as follows: the eye appeared to present with symptoms of epiphora; the pus reflux was visible when the lacrimal sac was compressed; and the lacrimal passage irrigation was obstructed, which was accompanied by reflux of secretions [13].

**Figure 2:**
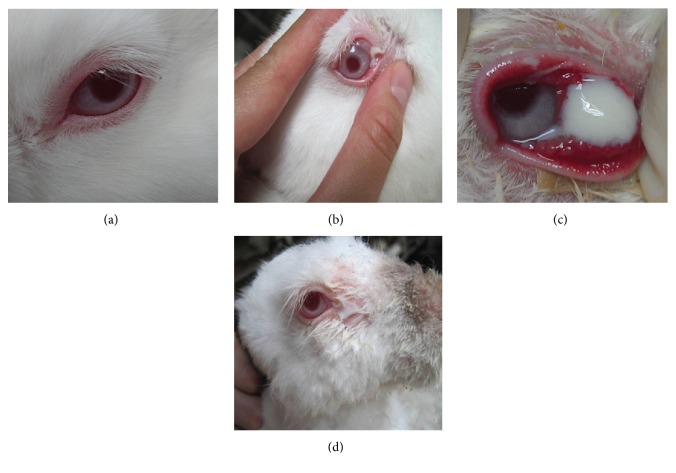
Representative photos taken in ophthalmological examination of the controls (a) and the 0.15 ml mixed resin injection group at 7 days (b), 7–30 days (c), and 30–90 days (d) after the injection.

After receiving an injection of self-curing resin, all animals were consecutively observed for 90 days. In the control group, the epiphora was invisible, hyperemia was not found in the conjunctiva, no mucus or purulent discharge had outflowed from the lacrimal punctum, and the lacrimal passage was unobstructed (Figure 2(a)), which meant that animals in the control group did not show evidence of chronic dacryocystitis.

In addition, chronic dacryocystitis was not found in either the 0.05 ml or the 0.1 ml self-curing resin injection groups. However, in the 0.15 ml resin injection group, 8 rabbits showed manifestations of chronic dacryocystitis at 7 days postinjection, which was evident by observation of an obstructed lacrimal passage and fluid reflux that was accompanied by a small amount of discharge (Figure 2(b)). From 7 to 30 days postinjection, hyperemia was gradually aggravated at the palpebral margin and conjunctiva, conjunctival sac discharge was increased, and there were significant resistance in lacrimal irrigation that was accompanied by viscous and purulent fluid reflux in all 8 rabbits (Figure 2(c)). From 30 to 90 days after injection, hyperemia at the palpebral margin and conjunctiva was slightly mitigated. Additionally, hair surrounding the lacrimal sac was shred, and there was still evidence of a viscous and purulent discharge in lacrimal irrigation (Figure 2(d)). Meanwhile, the remaining three rabbits in the 0.15 ml resin injection group did not show any evidence of epiphora and appeared to show lacrimal patency, which meant that they did not suffer from chronic dacryocystitis.

### 3.2. CT Dacryocystography

**Figure 3:**
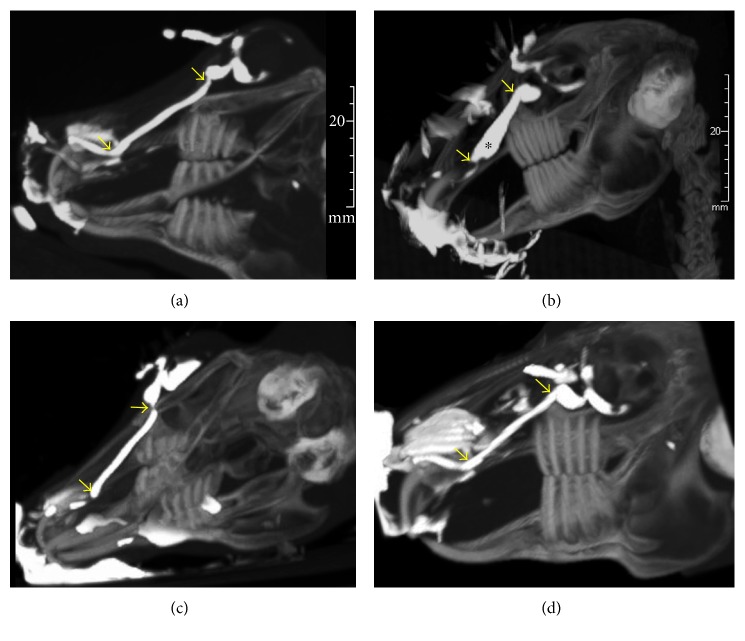
Representative MIP images in CT dacryocystography of the control group ((a), *n* = 11), the 0.15 ml treated group ((b), *n* = 11), the 0.10 ml treated group ((c), *n* = 11), and the 0.05 ml treated group ((d), *n* = 11). Yellow arrow indicates physiological curvatures, and ∗ indicates dilation.

Dacryocystography MIP images of the control group revealed that there were two physiological curvatures in the nasolacrimal duct of the normal rabbits, where the first one was located at the inlet of the nasolacrimal duct, namely at the junction of the lacrimal sac and the upper segment of the nasolacrimal duct. By contrast, the second curvature was at the junction of the nasolacrimal endosseous and intranasal segments (Figure 3(a)). The angle between the lacrimal sac and nasolacrimal duct inlet was approximately 90°, while that between the nasolacrimal endosseous segment and intranasal segment was about 130°.

**Figure 4:**
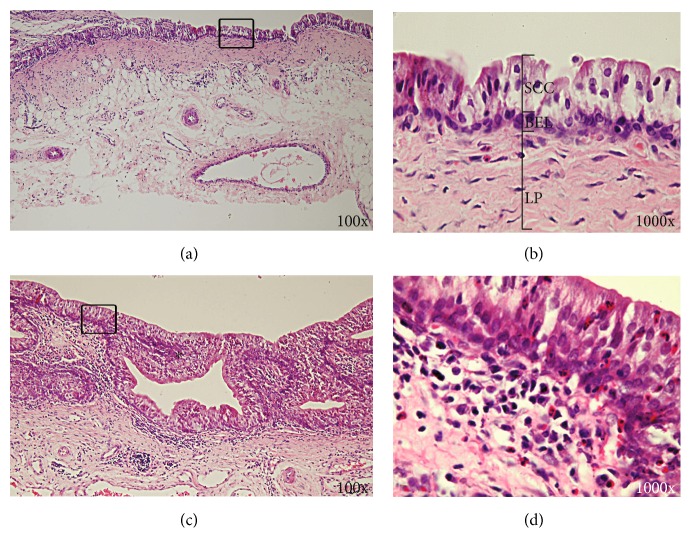
H&E stained histopathology images of the lacrimal sac from the control group (a and b) and the group with chronic dacryocystitis (c and d). (b) and (d) are the locally enlarged images of the rectangular selection on (a) and (c), respectively. SCC: superficial columnar cells; BEL: basal epithelial layer; LP: lamina propria. ∗ indicates the irregular papillary proliferation in the mucosal epithelium.

Following CT dacryocystography for chronic dacryocystitis, it was found that the rabbits in the 0.15 ml resin injection group had obstruction of the lower segment of the nasolacrimal duct, where it was observed to be close to the curvature of the nasolacrimal endosseous and intranasal segment. By contrast, the lacrimal duct that was located above the obstructed surface was significantly dilated and expanded compared with the contralateral lacrimal duct (Figure 3(b)). In these chronic dacryocystitis rabbits, the diameter at the widest segment of the nasolacrimal duct was as high as 5.85 ± 0.17 mm due to obstruction, which showed significant differences as compared with control rabbits. Moreover, the diameters at the widest segment of the nasolacrimal duct in the 0.05 and 0.1 ml resin injection groups with failed disease modeling (Figures 3(c)-4(d)) were 2.15 ± 0.12 mm and 2.18 ± 0.10 mm, respectively, which did not show any significant differences as compared with the control group (Table 1).

### 3.3. Pathological Changes of the Lacrimal Sac in Rabbits with Chronic Dacryocystitis

In controls, histological structures of the lacrimal sac wall were similar to those found in humans, where in the epithelial layer was composed of superficial columnar cells and deep basal epithelial layer. Under the epithelial layer is the lamina propria. Lymphocytes were scattered and/or clustered into lymph follicles in the propria layer. Meanwhile, collagen fibers and elastic fibers were visible in the propria layer, while small blood vessels were interconnected to form vascular plexuses, which were similar to cavernous-like structures (Figures 4(a) and 4(b)).

By contrast, lacrimal sacs from rabbits with successful modeling were subjected to routine pathological examination, which showed that inflammatory cell infiltrates were located in the lacrimal sac mucosa. In addition, irregular papillary proliferation was found in the mucosal epithelium, submucosal fibroblast proliferation and many other chronic inflammatory changes (Figures 4(c) and 4(d)).

## 4. Discussion

In this study, we successfully developed chronic dacryocystitis in rabbits by producing NLDO with intralacrimal injection of self-curing resin. Upon establishing the NLDO model, we observed a high incidence of chronic dacryocystitis.

Preparation of LDOD animal models has always been a recognized difficulty in relevant basic research. In terms of animal selection, Frame and Burkat [11] reported anatomical and histological differences of the rat, rabbit, pig, goat, and an additional 12 mammals as compared to humans, and thereby laid the foundation for preparing lacrimal duct models.

Of these proposed models, although the mouse is one of the most common animals in ophthalmic research, its lacrimal duct histology differs markedly from that of humans. Meanwhile, although the rabbit only has one lacrimal punctum and canaliculus, the lengths and structures of its lacrimal sac and nasolacrimal duct that locates at the posterior part are similar to those found in human physiology, and its lacrimal duct histology is also similar to the human lacrimal duct in some respects. For example, the lacrimal sac and nasolacrimal duct of both humans and rabbits are coated with multiple layers of columnar or pseudostratified columnar epithelial cells, with distributed mucus-secreting epithelial cells found in the spaces. Also, subepithelial structures of the rabbit are similar to those of humans, where the subepithelial structure is the adenoid layer and represents the fibrous connective tissue. By contrast, there is a distinct absence of a subepithelial adenoid layer in the intranasal segment. Thus, the rabbit has become the most widely used animal in studies of the lacrimal duct.

In terms of dacryocystitis modeling, others have prepared a chronic dacryocystitis rabbit model by subcutaneous injection of albumin followed by an injection of *Staphylococcus aureus* into the lacrimal sac and obtained a success rate of approximately 55 percent in a 6-month observation study [14, 15]. The principle was to inject albumin to cause lacrimal sac sensitization, mucosal edema, and temporary obstruction of lacrimal duct and then to inject bacteria that would provoke lacrimal dacryocystitis. However, the rabbits that were sensitized to albumin did not develop chronic inflammation without bacterial infection. Therefore, we believed that bacterial infection was the precondition to cause dacryocystitis. However, in our preexperiment, we injected 0.3 ml of 10^7^ cells/ml of a *Staphylococcus aureus* suspension liquid alone into the lacrimal duct. Following this study, no animal developed chronic dacryocystitis (data not shown). Thus, we speculate that drainage disorders caused by lacrimal stenosis or occlusion were also one of the necessary preconditions that caused chronic dacryocystitis.

Self-curing resin, also named room temperature-curing denture base resin, is a mixture of dental base acrylic resin liquid (mainly component is methyl methacrylate, MMA) and dental base acrylic resin powder (mainly component is poly methyl methacrylate, PMMA). At room temperature, MMA is a colorless transparent liquid. It is soluble in organic solvents, slightly soluble in water, and is prone to addition polymerization. MMA and PMMA are common biomedical materials for fast dental prosthesis in stomatology, with very stable chemical properties [16, 17]. The polymerization process of MMA and PMMA can be divided into the following six phases: (1) wet sand period: MMA has not yet penetrated into PMMA but exists among the PMMA particles. It appears that the liquid is much more than the powder. During this time period, the mixture has small blend resistance, no viscosity, and the touch feeling of wet sand. (2) Paste-like period: the surface of the powder is gradually swollen by the water with highly squeezed particles. There seems to be extra water getting out of the surface of the mixture. The mixture has no blend resistance. (3) Sticky silk period: the powder continues to be swollen, and the particles are further combined into a sticky piece. In this phase, the mixture tends to form ropiness and is sticky to the fingers and instruments. (4) Dough-like period: it is also known as plastic period. In this period, MMA is completely combined with PMMA. There is no extra MMA liquid. The mixture appears plastic dough without stickiness. (5) Rubber-like period: the surface of the mixture forms a hard scab after the water evaporation. Meanwhile, the inside of the mixture is becoming a hard and flexible rubber-like pattern. (6) Hardening period: the mixture becomes hard gradually with the evaporation of MMA liquid. The timing for each step needs to be strictly controlled because the process is influenced by the temperature. The time is shortened with the temperature increasing. Usually, it takes around 20 minutes from the mixing of MMA and PMMA to the formation of plastic period under the room temperature, and it lasts about 5 minutes for the plastic period.

Based on our earlier in vitro experimental results, we mixed dental base acrylic resin powder and dental base acrylic resin liquid (volumetric ratio of 5 : 2, or a weight ratio of 2 : 1) and blended this in a porcelain cup to a pasty state, at which point the mixture had good flowability and nonropiness and was nonadherent to utilities and was easy to inject into the lacrimal duct. If the mixture was injected at an earlier stage, it had an excessively large flowability. On the other hand, if the mixture was injected in a later stage, the mixture was in a state of ropiness and adhered to the utilities. Thus, by injecting the pasty mixture would prevent it from flowing out from the nasal cavity due to an excessively watery state and also would prevent it from difficult extraction and injection into the lacrimal duct due to an excessively thick state.

The time taken from blending the powder and liquid agents to a pasty state was affected by temperature, where a high temperature would shorten the time and vice versa. It would normally take about 30 s at room temperature of 25°C. Subsequently, a certain amount of the pasty substance was drawn into a number 9 lacrimal cannulated needle and was then injected into the lacrimal duct via the lacrimal punctum. We found that rabbits did not present with symptoms or signs of nasolacrimal duct obstruction if they received an injection of the self-curing resin with an amount less than 0.15 ml. This observation might be associated with both the lacrimal duct volume and compensatory features of the test animals themselves.

Injection with an amount less than or equal to 0.1 ml was unable to obstruct most of the cross-sectional area of the nasolacrimal duct, which was also confirmed by CT dacryocystography. Meanwhile, when the injection quantity reached 0.15 ml, many of the animals presented with chronic dacryocystitis, and the injected resin was often found to block the area around the angle between the endosseous and intranasal segments. Modeling was shown to fail in some rabbits receiving a sufficient injection volume. It is possible that this was due to an excessively high and fast rate of pressure that was applied when injecting materials into the lacrimal duct, which thus caused most of the materials to flow away from the nasal cavity. Anatomy also revealed that there was a low quantity of materials adhering to the nasolacrimal duct wall, which was insufficient to obstruct most of the cross-sectional area of the nasolacrimal duct. Three months after the model was successfully made, an obvious dilation of nasolacrimal duct was observed, which may be related to the weakness of bone nasolacrimal duct in rabbit. With time increasing, the segment above the obstruction will be passively expanded. The similar changes can also be observed in clinical settings. In children with long-term chronic dacryocystitis, the lacrimal sac and nasolacrimal duct above the obstruction are significantly dilated. In adults with chronic dacryocystitis, however, the nasolacrimal duct is usually not markedly dilated due to the hard bone structures while the lacrimal sac can be significantly expanded.

At present, the pathogenesis of LDOD is not clear. Janssen et al. suggested that the stenosis of the bone lacrimal duct is one of the most important causes of nasolacrimal duct obstruction [18]. Meanwhile, the other authors proposed that temporary obstruction of the nasolacrimal duct is firstly occurred due to the mucosal congestion and edema, resulting from ocular or nasal inflammations. And then, the repeated inflammation causes the irreversible fibrosis changes in mucosal and submucosal tissue structures of the nasolacrimal duct, finally resulting in the permanent obstruction or function loss of the involved nasolacrimal duct [19, 20]. In this study, the mechanical obstruction of the nasolacrimal duct was created by injecting the resinous polymer into the physiological transition of the nasolacrimal duct in rabbits. The results showed that the experimental rabbits had a chronic dacryocystitis, presenting with mucosal thickening, fibrous tissue deposition, and inflammatory cell infiltration, which can also be observed in the clinical settings. In the next experiment, a comparative study would be designed to investigate the pathological changes of the lacrimal mucosa at the different phases between the modeling rabbits and the patients who undergo DCR.

Some investigators have postulated that bacteria would accumulate and propagate at the lacrimal sac after obstruction of the nasolacrimal duct, resulting in dacryocystitis. However, following nasolacrimal duct obstruction, some patients present with dacryocystitis while others do not, although the specific reasons remain unclear. Clinically, we utilized endoscopy to observe the lacrimal duct of patients who did not develop dacryocystitis and found that in fact, there was evidence of inflammatory manifestation in the lacrimal sac mucosa and nasolacrimal duct mucosa (such as mucosal congestion and edema, as well as formation of fiber membrane in some cases); however, there was no purulent discharge. Meanwhile, in rabbits, we found that animals would suffer from chronic dacryocystitis once nasolacrimal duct obstruction was successfully modeled. Also, Gram-negative pasteurella and some other species of bacteria were detected in secretion cultures (data not shown). Thus, we speculated that there may be more conditioned pathogens in lacrimal passage of rabbits than in humans, which may cause dacryocystitis after obstruction. The hypothesis needs validation in the future.

## 5. Conclusions

In conclusion, the model described in this study is convenient, has a high success rate, and is able to better simulate the clinical manifestations of chronic dacryocystitis. It is expected to be applied in the study of the pathophysiology of the lacrimal duct and the pathogenesis of chronic dacryocystitis. However, this model has some defects and some aspects of this model require further investigation. For example, after people suffer from nasolacrimal duct obstruction, some of them develop chronic dacryocystitis; however, others do not appear to go on to develop dacryocystitis in the long term. However, in the current model described herein, once the lacrimal duct was completely obstructed, all the animals presented with dacryocystitis. Further studies are needed to clarify whether this is because rabbits inherently display more conditioned pathogens or whether there are alternative and as yet undiscovered biological mechanisms that are involved.

## Figures and Tables

**Table 1:** Comparison of the diameter at the widest segment of the nasolacrimal duct (measured in CT dacryocystography) among different groups.

Group	Number of cases	Diameter at widest segment (mm)	*P* value (compared with control group)
Control	11	2.11 ± 0.10	—
0.05 ml mixed resin	11	2.15 ± 0.12	0.57
0.1 ml mixed resin	11	2.18 ± 0.10	0.16
0.15 ml mixed resin	8	5.85 ± 0.17	0.001
